# Research integrity and transparency in CAM journals: an analysis of publication bias and reporting practices (1995–2023) in four leading journals

**DOI:** 10.1186/s41073-026-00221-2

**Published:** 2026-06-06

**Authors:** Stephen Neilson, Kevin Smith

**Affiliations:** Faculty of Applied Sciences, Department of Built Environment and Life Sciences, Abertay University, Dundee, Scotland DD1 1HG UK

**Keywords:** Alternative medicine, Complementary medicine, Integrative medicine, Journal analysis, Positive outcomes, Publication bias

## Abstract

**Background:**

Complementary and alternative medicine (CAM) remains widely used worldwide, yet longstanding concerns persist regarding the balance and reliability of the evidence presented in CAM journals.

**Objective:**

To examine long-term trends in publication practices within leading CAM journals, with particular attention to changes in publication types and the prevalence of positive versus negative study outcomes as indirect indicators of potential publication bias.

**Methods:**

We conducted a complete census of articles published in four leading CAM journals at two contemporary time points (2018 and 2023), replicating the design and classification framework of a seminal 2001 analysis covering 1995 and 2000. Articles were categorised by publication type, subject area, and author-reported study outcome (positive, negative, or inconclusive, corresponding to the “open” category used in the original 2001 study). Descriptive analyses were used to compare trends over time and with earlier findings.

**Results:**

The total number of published articles increased substantially between the earlier and contemporary periods. The proportion of empirical studies, particularly clinical trials, rose over time. However, the prevalence of positive outcomes also increased markedly, with positive findings accounting for over 80% of published articles in the contemporary period, compared with 49% in the earlier study. Negative and inconclusive outcomes remained relatively infrequent.

**Conclusions:**

Despite growth in publication volume and a shift toward empirical study designs, CAM journals continue to exhibit a pronounced predominance of positive outcome reporting. These findings suggest that longstanding concerns regarding publication bias in CAM publishing have not diminished over time and appear to have intensified, with selective publication and related reporting and dissemination practices plausibly contributing to the observed patterns. This has important implications for research integrity and evidence-based decision-making in medical practice.

## Introduction

In 2001, a significant paper was published that provided a detailed analysis of the publication practices of major journals specialising in complementary and alternative medicine (CAM) [1]. This analysis, spanning 1995 to 2000 and focusing on four leading peer-reviewed research-oriented journals in CAM, identified several problematic features in these publications. These included a predominance of positive findings, a reliance on surveys over clinical trials, and concerns regarding methodological rigor. These findings raised critical questions about the scientific integrity of CAM journals and their role in shaping public and professional perceptions of CAM. The 2001 paper's authors suggested that these problems may be due to CAM research being a young and rapidly growing field grappling with issues of bias and scientific maturity, and they expressed hope that improvements in research and publication standards would follow through time.

Bibliometric analyses have identified over 600 distinct forms of complementary and alternative medicine (CAM), with thousands of new CAM-related articles published annually, primarily in specialised journals. Notably, over 90% of these publications originate from a small number of journals, raising concerns about concentrated dissemination [2–4].

CAM research often suffers from significant methodological weaknesses, with many studies reporting positive outcomes despite the low prior probability of effectiveness for most CAM modalities. Critical analyses, including systematic reviews of various modalities, have highlighted major issues such as flawed data interpretation and a lack of compelling evidence for efficacy. Common methodological deficiencies include inadequate control groups, underpowered sample sizes, undisclosed multiple testing, inappropriate statistical methods, and unsupported conclusions [5–8].

Evaluating the rigor and objectivity of CAM research remains highly relevant, as persistent publication bias and overstated claims of efficacy could contribute to inappropriate use of CAM therapies in clinical practice. It is unknown whether CAM journals have improved in rigor or balance of findings over the past decades. Accordingly, the present study sought to revisit the findings of the original paper (hereafter referred to as “the 2001 study”), almost three decades after the initial census point (1995). To ensure comparability, we adopted essentially the same methodology as that study, examining the same four key journals and covering the years 2018 and 2023. In this paper, we examine whether, and to what extent, publication practices have improved or whether the same issues persist.

The aim of this study was to replicate the original analysis of CAM journals using data from 2018–2023 in order to determine what changes, if any, have occurred in publication types and outcome positivity over the intervening almost three decades.

## Methods

Articles published in the four journals in 2018 and 2023 were identified through searches of the Web of Science database, covering the four journals that were the subject of the 2001 study, namely: *Complementary Medicine Research* (*formerly Research in Complementary Medicine*, renamed in 2017), *Complementary Therapies in Medicine, Alternative Therapies in Health and Medicine, and Journal of Alternative/Integrative and Complementary Medicine* (formerly Journal of Alternative and Complementary Medicine renamed in 2021).

The years 2018 and 2023 were chosen to provide a recent 5-year interval for comparison with the earlier data (1995 and 2000), maintaining consistency with the design of the original study while extending the timeframe by more than two decades. The study did not involve sampling but rather comprised a complete census of all articles published in the four journals during the selected years. This approach ensured that the dataset fully represented the publication output for those time points.

Inclusion criteria mirrored those of the original study. All records retrieved in February 2024 were screened to exclude editorials, letters, corrections, and other non-primary content. For each included article, data were extracted and categorised into pre-defined categories, including publication type, subject area, and study outcomes. Outcomes were classified as positive (indicating CAM efficacy), negative (indicating no evidence of efficacy or favouring controls), or inconclusive (termed “open” in the original 2001 study), referring to ambiguous or mixed findings. Articles lacking statistical significance were classified based on author conclusions. This approach allows for direct comparison with the earlier findings.

While the core approach to paper type categorisation followed the 2001 study to enable comparability, minor adaptations were made. Two additional subject categories, *Traditional Chinese Medicine* and *acupuncture*, were introduced to reflect the substantial number of recent articles focusing on these topics. Where articles could reasonably fit multiple subject categories, classification was based on the predominant focus as judged from the abstract.

When extracting the earlier data, minor inconsistencies were identified in the tabulated values reported in the 2001 study, where some totals did not correspond to the sum of their constituent categories. For the purposes of the present analysis, totals were recalculated where necessary to ensure internal consistency. A small discrepancy in the 1995 ATHM data could not be fully reconciled with the published figures and is therefore indicated in Table 1.

**Table 1:**
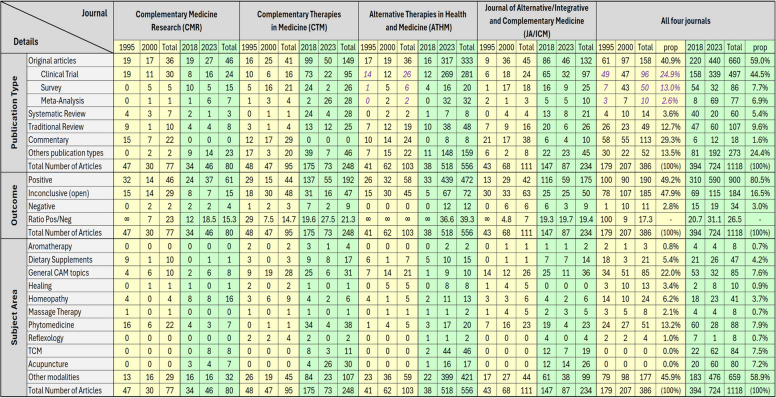
Publication types, outcomes, and subject areas in four CAM journals: comparison of 1995/2000 and 2018/2023

Data for 1995 and 2000 are reproduced from the original 2001 study. Minor inconsistencies were identified in the tabulated values of that study, where some totals did not correspond to the sum of their constituent categories. In Table 1 these totals have been recalculated to ensure internal consistency. One small discrepancy in the 1995 data for Alternative Therapies in Health and Medicine (ATHM), concerning the distribution of original-article subtypes, could not be fully reconciled with the published totals; the affected values are therefore shown in italics and retained as reported in the original source

The columns labeled ‘prop’ show the proportions (as percentages) of each row’s value relative to the Total Number of Articles for each of the three domains

Initial screening and classification of eligible articles were performed by one researcher. To enhance consistency, a second researcher subsequently reviewed all inclusion decisions and data categorizations. As the eligibility assessment and categorisation were based on clear, objective criteria (publication year, article type, and author-reported conclusions), full independent duplicate screening was not deemed necessary.

Consistent with the 2001 study’s exploratory scope, we did not perform a formal quality appraisal of each article (e.g., risk of bias assessment). Instead, study design and outcome reporting serve as indirect indicators of methodological quality and bias.

### Data flow and PRISMA diagram

**Fig. 1:**
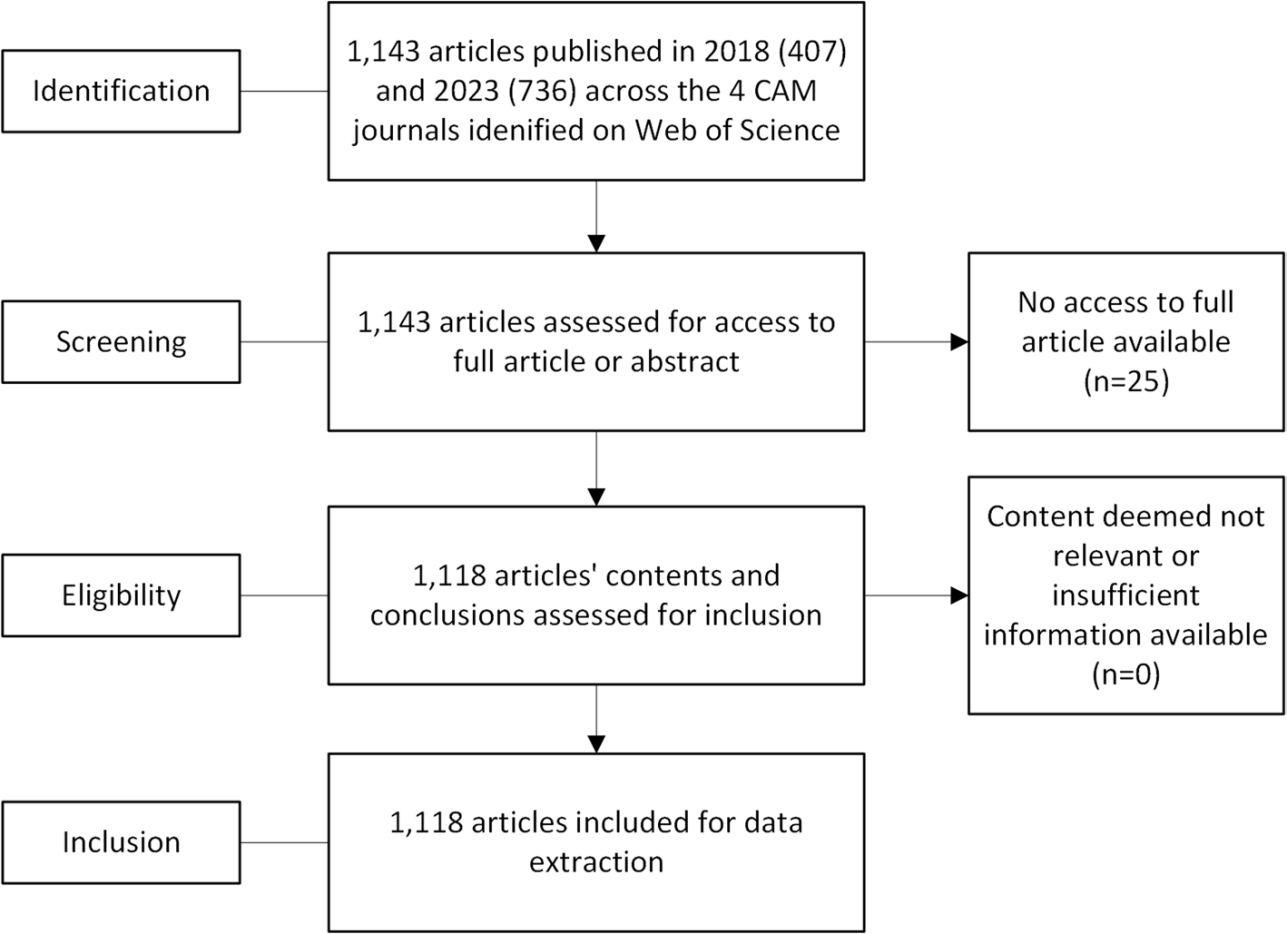
Flow diagram of article selection

Figure 1 illustrates the flow of study selection: 1143 records were identified (407 for 2018 and 736 for 2023); after excluding articles for which no access to full text was available (25) and the content of which was deemed not relevant or insufficient information available (0), we included 1118 articles for analysis.

### Outcomes

The primary outcomes were (i) the proportion of articles reporting positive outcomes versus negative or inconclusive outcomes, and (ii) the distribution of article types (e.g., clinical trials, surveys, meta-analyses) within each time period. The principal aim was to compare these outcomes between the earlier period (1995–2000) and the recent period (2018–2023) to assess changes over time. Secondary metrics included changes in the prevalence of other publication categories (such as reviews, commentaries, and case studies) and trends in subject areas reported.

### Statistical methods

All analyses were descriptive and consistent with the methodology of the 2001 baseline study. Each article was classified by publication type, subject area, and overall outcome (positive, negative, or inconclusive) based on its abstract. Classification criteria matched those of the baseline study, with the addition of categories for Traditional Chinese Medicine and acupuncture, which had become more prominent.

Article counts were tabulated separately for each journal and year. Totals were then aggregated across all journals for 2018 and 2023, and combined to generate overall figures for the 2018–2023 period, enabling comparison with the earlier data from 1995–2000. Percentages were calculated by dividing the count in each category by the total number of articles for the relevant year or combined period. Percentages are reported to one decimal place in Table 1, while values cited in the text are rounded to the nearest whole number for clarity.

The positive-to-negative outcome ratio was determined by dividing the number of positive outcome articles by the number of negative outcome articles. Articles classified as inconclusive were excluded from this ratio. A higher ratio indicated a greater predominance of positive findings.

No inferential statistical tests were performed. Given that the data represented a complete census of all eligible articles, and that the study was descriptive and exploratory in nature, hypothesis testing (e.g., chi-square comparisons) was not considered appropriate. Results are presented as observed frequencies, percentages, and ratios to characterise trends over time.

### Open science

Data extraction and analysis were carried out using standard, widely available tools. Articles were retrieved from the Web of Science database (Clarivate), and article counts and classifications were manually tabulated in Microsoft Excel (Microsoft Office 365). No specialised statistical or bibliometric software was required, as the analyses were descriptive and involved only simple counts and proportions. Figures were prepared using standard office software.

No prospective protocol or registry was filed for this retrospective analysis of published literature. Our methods closely follow those of Schmidt et al. [1], and any modifications (for example, adding new subject categories for Traditional Chinese Medicine and acupuncture) are explicitly noted in the Methods. Because this work is a follow-up bibliometric review of existing publications rather than a new clinical trial or prospective study, a formal pre-registered protocol was not applicable.

## Results

Table 1 details in numerical terms the changes between the 2001 study and the present study. Totals in the 2001 study refer to the combined data from the census points of 1995 and 2000, and totals for the present study combine data from the census points of 2018 and 2023. Percentage values are reported to one decimal place in Table 1; values cited in the text are rounded to the nearest whole number where appropriate for ease of presentation. The main findings, plus some additional ones, are described below.

### Main findings

Across all four journals, the total number of articles increased substantially, rising from 386 in the 2001 study to 1118 in the present study. This growth was reflected consistently across the individual census points: 179 articles in 1995, 207 in 2000, 394 in 2018, and 724 in 2023. This increase in total indicates a major expansion of CAM literature, reflecting the growing academic interest in CAM [9]. It should also be noted that since the 2001 study, at least four additional peer-reviewed general CAM journals that focus on research have been published,^1^ further reflecting this feature of expanding numbers of CAM publications. (To ensure comparability with the 2001 results, these newer journals were not included in the present study).

However, context is important here: global scientific publication output across all fields has been growing at approximately 4–5% annually – equating to a doubling every 15–17 years [10], implying a 23-year increase of roughly 270% over the time period between the 2001 study and the present study. This indicates that the publication growth of the four CAM journals has been broadly in line with that of the overall scientific literature.

**Fig. 2:**
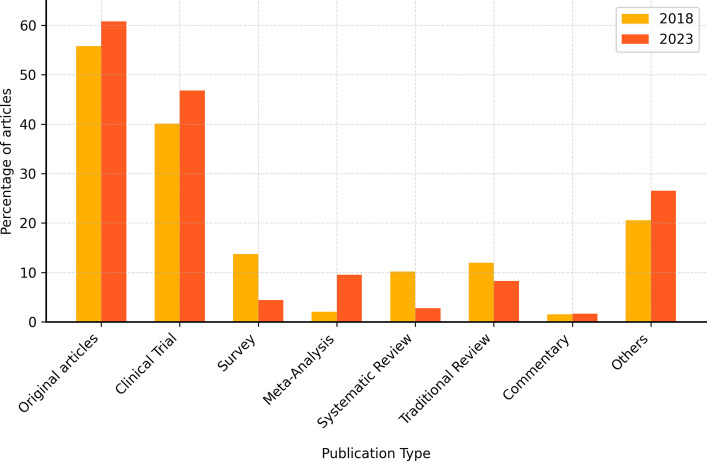
Distribution of publication types by year

In the 2001 study, the proportion of original articles – defined as clinical trials, surveys, and meta-analyses – rose from 34% in 1995 to 47% in 2000. This upward trend continued in the present study, with original articles comprising 56% in 2018 and 61% in 2023. However, in the 2001 study, the increase was largely due to a six-fold increase in the number of published surveys. By contrast, our results show that clinical trials had the most substantial increase, rising from 25% of all articles in the 2001 study to 44% in the present study. This is in contrast to the trend within the 2001 study itself, where the proportion of clinical trials declined from 27% in 1995 to 23% in 2000, a 4% decrease. Figure 2 summarises changes in publication types observed in the present study (2018 and 2023).

The authors of the 2001 study attributed the 4% decline in the proportion of clinical trials to national initiatives (including in the US, Germany, Switzerland, and Italy) prioritising large-scale CAM clinical trials, leading to increased funding and attracting researchers with conventional medical backgrounds. They suggested that these researchers likely favoured mainstream medical journals, which offer greater readership and recognition, resulting in more clinical trials being published there and fewer in CAM journals.

Since 2001, funding for CAM research has plateaued. In the United States, the National Center for Complementary and Integrative Health (NCCIH), formerly NCCAM, has seen relatively stable funding levels in real terms, with no substantial increases over time [11]. In Europe, funding for CAM research is less centralised, and there is no evidence of a significant overall increase in resources [12]. It is possible that such limited resources mean that much of the clinical research in CAM is being conducted by CAM-focused researchers who direct their work toward CAM-specific journals. This dynamic may be contributing to the observed increase in both the absolute number and proportion of clinical trial papers in CAM journals reported in the present study.

**Fig. 3:**
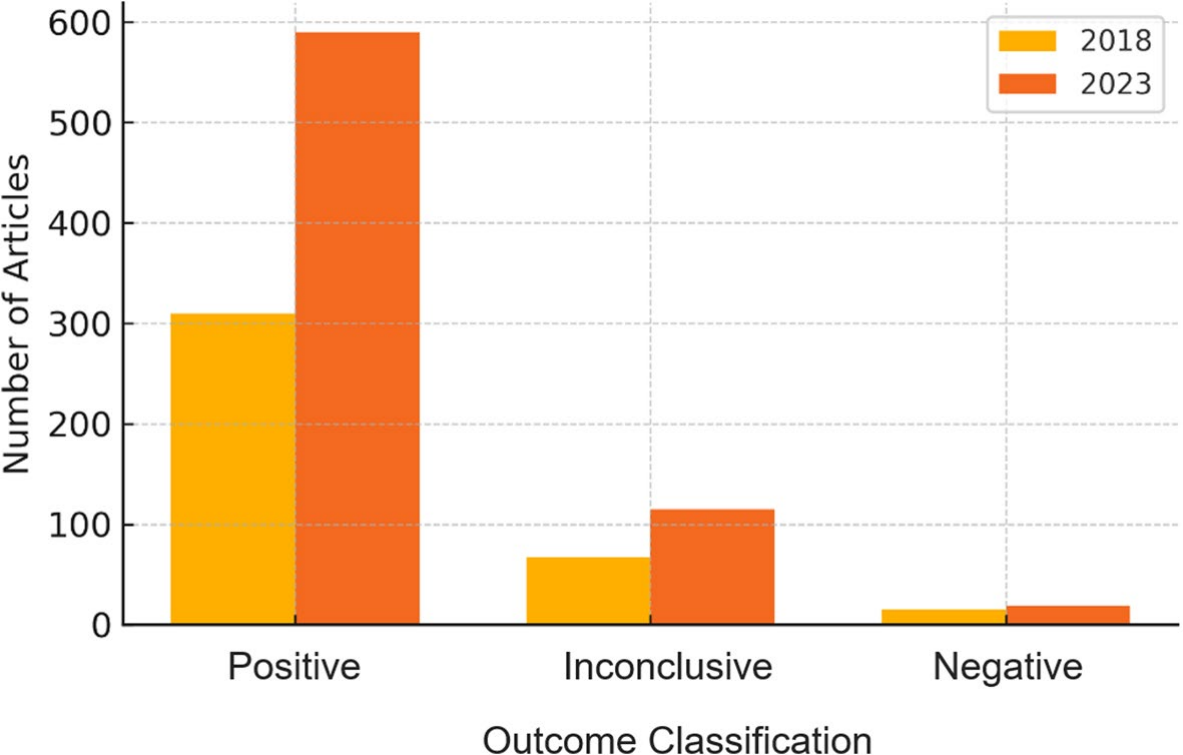
Outcome classification by year

Figure 3 provides an overview of the direction of outcomes. The 2001 study found fewer positive and more negative or inconclusive outcomes in 2000 compared to 1995, with the positive-to-negative ratio dropping from 100 to 9. Nevertheless, the average ratio for the two census points was high, at 17.3. In comparison, the present study reported an increased ratio across its census points, at 20.7 (2018) and 31.1 (2023). Overall, there was a marked increase in positive outcomes, with positive articles comprising 81% of total articles compared to 49% in the 2001 study. The positive-to-negative ratio averaged 26.5 in the present study, indicating a substantial rise in reported positive findings. While the 2001 study was not specifically designed to investigate publication bias, its authors noted that the best available evidence – such as systematic reviews and meta-analyses of rigorous clinical trials – does not support the predominance of positive conclusions in CAM research [13]. They also reported evidence of location bias in controlled clinical trials of CAM interventions [14], raising the possibility that publication venue may influence the balance of positive and negative findings in the CAM literature.

However, the suggestion that the immaturity of the field – an issue expected to resolve over time – accounts for this bias is not supported by the findings of the present study. The substantial increase in the proportion of positive outcomes observed in the present study compared to the 2001 study demonstrates that the trend of predominantly positive reporting in CAM journals has not only persisted but intensified over time – despite the field presumably maturing in the near 30-year period involved.

### Additional findings

Beyond the above patterns observed in respect of clinical trials and outcome reporting, other notable shifts in publication types were evident. The proportion of meta-analyses increased modestly, from 3% in the 2001 study to 7% in the present analysis, although they remained a small fraction of total articles. Systematic reviews, which had more than doubled during the earlier period (rising from 4 to 10 articles), declined in absolute number between 2018 and 2023 (from 40 to 20), yet their overall representation remained similar at 5%. Traditional reviews fluctuated over time, decreasing slightly in the 2001 study but increasing again in the present period; overall, however, they accounted for a slightly smaller share of publications, dropping from 13 to 10%.

Surveys, which had previously expanded sixfold between 1995 and 2000, reversed this trajectory. Their proportion fell from 13% in the 2001 study to 8% in the current period, suggesting a move away from survey-based research. Commentaries showed an even sharper decline: once comprising nearly a third of all articles (29%), they have become almost negligible in recent years, now accounting for less than 2%.

In contrast, articles classified as ‘other’ – including case studies, in vitro experiments, and diagnostic techniques – grew substantially, increasing from 14 to 24% of total publications. Regarding subject areas, the scope of topics remained broadly similar across both studies. However, two new domains, Traditional Chinese Medicine (TCM) and acupuncture, had expanded sufficiently by 2018–2023 to warrant their own categories. Additionally, articles grouped under ‘other modalities’ (e.g., aromatherapy, dietary supplements, homeopathy, massage therapy, phytomedicine) rose as a proportion of total publications, increasing from 46 to 59%.

Taken together, these findings suggest that while certain forms of higher-level evidence, such as meta-analyses, have become more common, the overall composition of CAM publications has diversified, with a notable shift away from editorials and commentaries and toward a broader range of study types and topics.

## Discussion

This study examined whether publication practices in four major CAM journals have changed since the original 2001 analysis. The results show that while the total number of publications has increased markedly – mirroring growth in scientific output more broadly – the proportion of articles reporting positive outcomes has also risen substantially. Positive findings accounted for 81% of all publications in the current study, compared to 49% in the earlier period. Although a moderate expansion of clinical trials and meta-analyses was observed, the persistence and intensification of overwhelmingly positive results suggest that the publication bias highlighted by Schmidt et al. has not diminished over time.

The original authors proposed that this bias might reflect the field’s relative immaturity, anticipating that methodological rigor and more balanced reporting would emerge as CAM research matured. However, the current findings do not support this expectation. Despite nearly three decades of additional research activity, positive-to-negative ratios have increased further, reinforcing the concern that CAM journals may selectively promote studies favourable to CAM modalities. This pattern is consistent with broader critiques of CAM literature, which note that many therapies lack robust evidence of efficacy and are subject to selective reporting and publication bias.

Importantly, the observed positivity rates are striking when considered in the context of broader biomedical research. For example, a large analysis of clinical trials found average success rates of approximately 14% across mainstream medical interventions, ranging from 3.4% in oncology to 33% in vaccines [15]. The discrepancy between these benchmarks and the 81% positivity observed here underscores the plausibility that selective publication practices remain pervasive in the CAM field.

There are well-documented concerns in medicine that trials reporting positive findings are more likely to be published than those with negative findings – a phenomenon commonly described as study publication bias [16]. Other biases that suppress negative findings or amplify positive ones have also been reported, and can be particularly salient where oversight and methodological standards are variable. These include outcome reporting bias and interpretive ‘spin’ [16–18]. Whereas study publication bias involves the non-publication of an entire study, outcome reporting bias refers to selective non-reporting of outcomes within a published article, including omission of negative outcomes or the switching of non-significant primary outcomes with statistically significant secondary outcomes [16]. Both forms of bias pose a substantial threat to the validity of meta-analyses by distorting the evidence base on which they rely [19]. Even when non-significant results are reported, interpretation may still be framed in ways that exaggerate apparent efficacy. Such reporting practices, commonly referred to as spin, include emphasising statistically significant secondary analyses despite non-significant primary outcomes [17]. Experimental evidence indicates that spin can lead readers to perceive interventions as more effective than is warranted [20]. Empirical evidence that publication and related biases can inflate apparent treatment effects over time has been demonstrated across a wide range of clinical contexts, including a widely cited and influential analysis in depression [21], as well as notable empirical studies in oncology [22] and vascular surgery [23].

Related questionable research and reporting practices may further contribute to an excess of positive findings in the published literature. These include p-hacking, whereby analytic flexibility is exploited to obtain statistically significant results, and hypothesising after the results are known (HARK-ing), in which unanticipated findings are presented as if they were predicted a priori [7, 24–26]. Such practices can operate even in the absence of overt misconduct and may, over time, distort the apparent efficacy of interventions. In this context, the high positivity rate observed in the present study (81%) should be interpreted as a signal warranting continued scrutiny of publication practices and editorial standards within CAM-focused journals.

An additional mechanism that may be particularly salient in the present context is location bias, whereby more favourable CAM studies are disproportionately published within CAM-specialist journals. Empirical evidence for this phenomenon has been demonstrated in a systematic analysis of controlled clinical trials of complementary therapies, which found a marked predominance of positive outcomes in CAM journals, particularly those without impact factors, alongside significantly poorer methodological quality among positive trials compared with negative ones [14]. By contrast, high-impact mainstream medical journals published roughly equal numbers of positive and negative CAM trials, suggesting that journal venue and editorial context materially shape the apparent evidential profile of CAM interventions. As discussed elsewhere, such patterns need not imply deliberate suppression of unfavourable findings, but rather reflect structural features of CAM research and publishing ecosystems, in which specialist journals may be more receptive to affirmative findings and less stringent in methodological appraisal [27]. Over time, this selective dissemination across publication venues may contribute to a self-reinforcing literature in which apparent efficacy is amplified and critical scrutiny is attenuated.

The apparent decline in commentaries and the increased proportion of empirical studies, including clinical trials and meta-analyses, might be viewed as signs of maturation and professionalisation of CAM publishing. However, the persistent imbalance in outcome reporting raises questions about whether these developments have translated into more critical appraisal of evidence or simply increased the volume of research with a positive framing. This dynamic carries implications for practitioners and patients who rely on CAM journals for information, as an inflated appearance of efficacy can distort decision-making and expectations. It is also worth noting that while meta-analyses are often regarded as authoritative forms of evidence, when primary studies are affected by methodological limitations or selective publication, the aggregation of such studies risks reinforcing rather than correcting distortions in the evidence base.

Accordingly, the present study’s finding of an 81% positivity rate – significantly higher even than the 49% reported in the 2001 study – underscores the risk that such a skewed representation could profoundly distort the perceptions of CAM among the readership of these journals.

### Limitations of the study

Several limitations should be acknowledged. First, although efforts were made to apply consistent inclusion criteria and classification procedures, the assignment of articles to specific categories involved some subjective judgement. Second, the reliance on broad "other" categories for publication types and modalities limited the granularity of the analysis. Third, the study focused exclusively on four journals previously examined in 2001, omitting other established CAM journals that have emerged since then. While this restriction was necessary to maintain comparability over time, it means the results may not fully represent the broader CAM publishing landscape. Finally, this study cannot directly measure publication bias, as it did not compare published results to unpublished studies; inferences about bias are based solely on observed patterns and prior literature.

## Conclusions

In summary, our study demonstrates that despite the passage of almost three decades, the publication practices of four leading CAM journals have not progressed toward a more balanced representation of evidence. The trend toward positive reporting has become more pronounced. While the growth in clinical trials and meta-analyses suggests greater research engagement, it also suggests the need for improved editorial standards and more transparent reporting of negative or inconclusive findings. Addressing these issues will be critical to aligning CAM literature with broader scientific norms and ensuring that practitioners and the public are presented with an accurate assessment of evidence in the domain of CAM.

## Data Availability

The analyses in this study are based exclusively on descriptive counts derived from publicly available journal articles. All extracted variables and summary counts are fully reported in the tables and figures within the manuscript, and no additional item-level data were generated beyond these classifications.

## Footnotes

1 *BMC Complementary Medicine and Therapies; Journal of Evidence-Based Integrative Medicine; Journal of Integrative Medicine; European Journal of Integrative Medicine.*
